# Prioritizing problems in and solutions to homecare safety of people with dementia: supporting carers, streamlining care

**DOI:** 10.1186/s12877-017-0415-6

**Published:** 2017-01-19

**Authors:** Lorainne Tudor Car, Mona El-Khatib, Robert Perneczky, Nikolaos Papachristou, Rifat Atun, Igor Rudan, Josip Car, Charles Vincent, Azeem Majeed

**Affiliations:** 1 Department of Primary Care and Public Health, School of Public Health, Faculty of Medicine, Imperial College London, London, UK; 2 Department of Global Health and Population, Harvard T.H. Chan School of Public Health, Boston, USA; 3 Neuroepidemiology and Ageing Research Unit, School of Public Health, Faculty of Medicine, Imperial College London, London, UK; 4 Department of Psychiatry and Psychotherapy, Technische Universität München, Munich, Germany; 5 Department of Global Health and Population, Harvard T.H. Chan School of Public Health, Boston, UK; 6 Centre for Global Health Research, Usher Institute of Population Health Sciences and Informatics, The University of Edinburgh Medical School, Edinburg, UK; 7 Centre for Population Health Sciences, Lee Kong Chian School of Medicine, Nanyang Technological University, Singapore, Singapore; 8 Department of Experimental Psychology, Medical Sciences Division, University of Oxford, Oxford, UK; 9 Lee Kong Chian School of Medicine, Nanyang Technological University, Singapore, Singapore

**Keywords:** Dementia care, Homecare, Priority-setting, Patient safety, Clinicians, Collective wisdom

## Abstract

**Background:**

Dementia care is predominantly provided by carers in home settings. We aimed to identify the priorities for homecare safety of people with dementia according to dementia health and social care professionals using a novel priority-setting method.

**Methods:**

The project steering group determined the scope, the context and the criteria for prioritization. We then invited 185 North-West London clinicians via an open-ended questionnaire to identify three main problems and solutions relating to homecare safety of people with dementia. 76 clinicians submitted their suggestions which were thematically synthesized into a composite list of 27 distinct problems and 30 solutions. A group of 49 clinicians arbitrarily selected from the initial cohort ranked the composite list of suggestions using predetermined criteria.

**Results:**

Inadequate education of carers of people with dementia (both family and professional) is seen as a key problem that needs addressing in addition to challenges of self-neglect, social isolation, medication nonadherence. Seven out of top 10 problems related to patients and/or carers signalling clearly where help and support are needed. The top ranked solutions focused on involvement and education of family carers, their supervision and continuing support. Several suggestions highlighted a need for improvement of recruitment, oversight and working conditions of professional carers and for different home safety-proofing strategies.

**Conclusions:**

Clinicians identified a range of suggestions for improving homecare safety of people with dementia. Better equipping carers was seen as fundamental for ensuring homecare safety. Many of the identified suggestions are highly challenging and not easily changeable, yet there are also many that are feasible, affordable and could contribute to substantial improvements to dementia homecare safety.

**Electronic supplementary material:**

The online version of this article (doi:10.1186/s12877-017-0415-6) contains supplementary material, which is available to authorized users.

## Background

In the UK, there are currently around 850,000 people with dementia [1]. While some reports show that the prevalence of the dementia in the UK is stabilising, others predict a rise to over 1 million by 2025 [1, 2]. The UK’s dementia expenditure currently amount to about £26.3 billion a year of which £11.6 billion is unpaid care, as the largest part of dementia patients’ care and costs are taken on by patients’ families [3, 4]. The social and healthcare services rely on carers’ to provide care to people with dementia [5].

Caring for dementia patients requires specific skills and knowledge, is physically and emotionally challenging and often leads to carers’ burnout [6–8]. A steady migration of medical devices and technologies into homes is placing an additional burden on carers [9].

Prior research on dementia care safety largely focuses on institutional rather than home settings (16). Yet homecare is more liable to patient safety incidents as homes are neither designed nor regulated like healthcare institutions. The annual rate of adverse events in homecare patients is 13.2%, one-third of which are considered preventable [10]. The Care Quality Commission, an independent regulator for health and social care in England, reported that almost a quarter of homecare providers fail to meet basic standards, leaving service users feeling “vulnerable and undervalued” [11]. Finding effective ways for supporting carers of people with dementia living at home and creating safe home environments is one of the top ten priorities for dementia research [12]. It is essential to proactively search for main safety concerns and their effective solutions rather that to wait to learn from tragic events. Clinicians, as important stakeholders in care of people with dementia, can help determine the dementia homecare safety priorities. In this study, we invited clinicians to identify main problems and solutions relating to homecare safety of people with dementia in North-West London.

## Methods

**Fig. 1:**
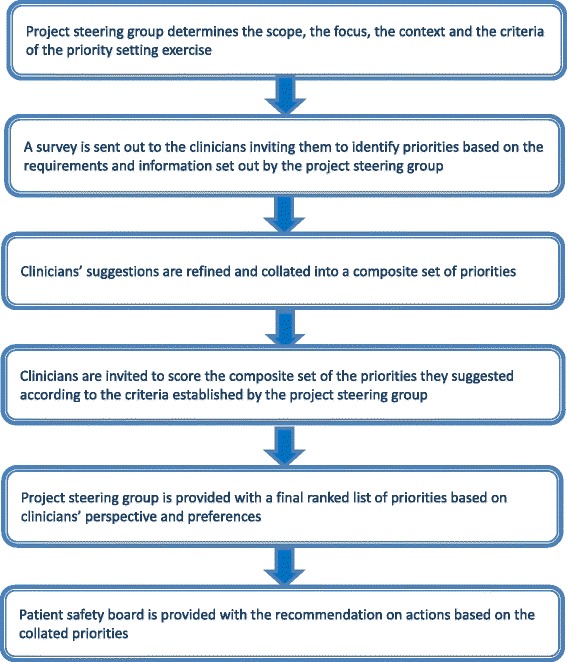
The PRIORITIZE methodology flow diagram

We developed and implemented the PRIORITIZE method, an adaptation of the Child Health and Nutrition Research Initiative (CHNRI) approach [13–15], to determine the main problems and solutions relating to homecare safety of people with dementia (Fig. 1).

Designed to reveal both the main problems and solutions for healthcare services delivery according to clinicians, the final output of PRIORITIZE is presentation of the top priorities categorized according to level of implementation: a) actions for clinicians b) actions for healthcare organisations and c) actions for health system custodians (Fig. 1). This study is a service evaluation as well as a quality and safety improvement initiative and therefore did not require ethics or governance approval according to the UK’s Health Research Authority guidance [16, 17]. The project steering group (Imperial College Health Partners’ Patient Safety Board) focused on homecare safety of people with dementia and established the most pertinent criteria to guide the prioritisation of the collated suggestions, i.e. scoring of problems and solutions (Table 1). This study is a part of a larger project aimed at determining clinician-identified priorities for patient safety in primary, cancer and dementia care [18–20].

**Table 1:** Scoring criteria

Problems	Solutions
Frequency: This patient safety threat is common Severity: This patient safety threat leads to high rates of mortality, morbidity and incapacity Inequity: This patient safety threat affects lower socio-economic groups or ethnic minorities more than other groups Economic impact: The consequences of this patient safety threat are costly to the healthcare system Responsiveness to solution: This incident is amenable to a solution within 5 years	Feasibility: The implementation of this solution is feasible Cost-effectiveness: This solution is cost-effective Potential for saving lives: This solution would save lives

In the first phase of the study, we developed an open-ended questionnaire for clinicians to identify the main problems and solutions relating to homecare safety of people with dementia. The questionnaire was piloted on a smaller sample of primary care physicians and trainees and amended accordingly. The final questionnaire was distributed in both paper-based and online versions and disseminated via email lists, snowballing (participants were asked to forward the survey to colleagues), and visits to general practices in North-West London (Additional file 1: Appendix 1). We targeted different healthcare professionals working with people with dementia such as GPs, nurses, social care professionals, occupational therapists and psychotherapists etc.

In the second phase, we created a prioritization matrix consisting of collated priorities and statements outlining prioritization criteria (Additional file 1: Appendix 2). We then invited clinicians to categorize the priorities according to the prioritization criteria using four options: score of 1 for ‘Yes - I agree with this statement’, score of 0 for ‘No - I do not agree with this statement’, score of 0.5 for ‘Unsure - I am unsure whether or not I agree’ and no score (blank) for ‘Unaware – I do not feel sufficiently familiar or confident to score this suggestion’ (Additional file 1: Appendix 2). As the scoring process took about an hour to complete, we offered a token payment to the participants in a form of a £50 voucher. From the initial cohort of dementia care clinicians, we arbitrarily invited participants to score the priorities.

The intermediate scores, i.e. scores for each criterion for every suggestion, were calculated by adding up all the answers (“1,” “0,” or “0.5”) and dividing the sum by the number of received answers. All intermediate scores for all research options are therefore assigned a value between 0 and 100. The overall priority score was then computed as the mean of the scores for each of the five criteria for problems and three for solutions. Higher ranked solutions received more “Yes” responses for each of the criteria and a higher score.

We were also interested in exposing the priorities that were considered important by most participants, i.e. suggestions with the greatest level of agreement among the clinicians. The Kappa statistic was deemed an inappropriate test in that sense within this methodology due to the sample size, the non-standardised categorical nature of data, the option of blank response to some statements and the number of our different criteria used for scoring. Instead, we evaluated the inter-rater agreement using the average expert agreement (AEA) [13]. The AEA is the proportion of scorers selecting the mode (the most common score) for each research question. AEA does not provide information on statistical significance of any differences between scorers, but is pertinent to decision makers as it gives an indication of the degree of agreement between clinicians in terms of priorities. The AEA was calculated using the following formula:

$$ \mathrm{A}\mathrm{E}\mathrm{A}=\frac{1}{5}{\displaystyle \sum_{\mathrm{q}=1}^5\frac{\mathrm{N}\left(\mathrm{scorers}\kern0.5em \mathrm{who}\kern0.5em \mathrm{provided}\kern0.5em \mathrm{the}\kern0.5em \mathrm{most}\kern0.5em \mathrm{frequent}\kern0.5em \mathrm{response}\right)}{\mathrm{N}\left(\mathrm{scorers}\right)}} $$

$$ \mathrm{A}\mathrm{E}\mathrm{A}=\frac{1}{3}{\displaystyle \sum_{\mathrm{q}=1}^3\frac{\mathrm{N}\left(\mathrm{scorers}\kern0.5em \mathrm{who}\kern0.5em \mathrm{provided}\kern0.5em \mathrm{the}\kern0.5em \mathrm{most}\kern0.5em \mathrm{frequent}\kern0.5em \mathrm{response}\right)}{\mathrm{N}\left(\mathrm{scorers}\right)}} $$

(where q is a question that experts are being asked to evaluate competing patient safety threats (in this case homecare safety threats), ranging from 1 to 5 for problems and 1 to 3 for solutions).

To analyse the proposed problems, we classified them using the following contributing factors to safety in home health care: system & organizational, home environment, carer-related (including both family members and unpaid carers as well professional carers), patient-related, healthcare provider-related. To analyse proposed solutions, we determined the main actors or settings they were intended for (i.e. carers, patients, healthcare providers, public, home environment or other services) and the type of the suggested intervention (education, organization of care, review & supervision, working conditions, recruitment & vetting, safety proofing).

## Results

**Fig. 2:**
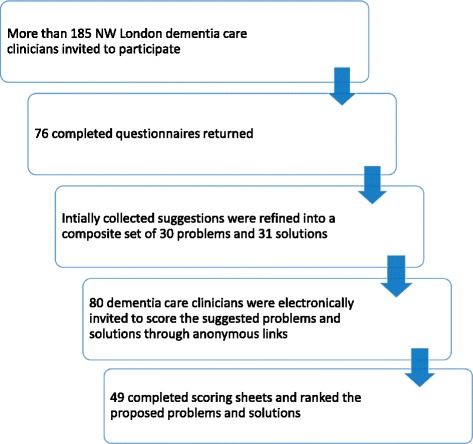
Participants’ flow diagram

More than 185 clinicians working in dementia care in North-West London were invited to participate in the first phase of the study. Most of the 76 (41%) completed questionnaires were answered by GPs and nurses (Additional file 1: Appendix 3). We initially collated 143 suggestions for homecare safety-related problems and 123 suggestions for solutions. As they were overlapping, these initial suggestions were grouped into a composite set of 27 distinct problems and 30 proposed solutions and ranked using the preselected criteria (Fig. 2).

The top three problems in homecare safety of people with dementia were reduced GP budgets, day centres’ and social care/services’ resources and carers’ lack of appropriate training and/or qualifications (Table 2). The top three solutions to homecare safety threats focused on involvement and education of family members as carer, training of carers on handling the patient and reviews of family carers to ensure they are coping (Table 3).

**Table 2:** Top ten problems leading to patient safety threats in homecare

RANK	Proposed problems leading to homecare safety threats	Total Priority Score	Type of the actor or setting related to homecare safety problems	Type of the contributory factor leading to homecare safety problems
1.	Reduced GP budgets, day centres and social care/services resources	82.7	System & organizational	Resources
2.	Professional carers lacking proper training and qualifications	81.6	Clinicians	Knowledge and skills
3.	Family carers lacking training and education	79.8	Carers	Knowledge and skills
4.	Patient neglecting themselves	78.4	Patient	Support
5.	Social isolation	78.2	Patient & Carers	Support & Relationship
6.	Patient forgetting to take the medication	77.1	Patient	Support
7.	Unsafe design of home environment	77.1	Home environment	Setting
8.	Poor mobility and falls	75.7	Patient	Support
9.	Family members unable to manage the patient	75.7	Carers	Knowledge and skills & Support
10.	Health deterioration in family members due to burden of caring	74.9	Carers	Support

(Clinicians scored problems using the following criteria: frequency, severity, inequity, economic impact and responsiveness to solution (Table 1). The scoring options were 1 for “yes (e.g. this problem is common)”, 0 for “no (e.g. this problem is uncommon)”, 0.5 for “unsure (e.g. I am unsure if this problem is common)” and blank for “unaware e.g. I do not know if his problem is common)”. Total Priority score is the mean of the scores for each of the five criteria and is ranging from 0 to 100. Higher ranked problems received more “Yes” responses for each of the criteria and a higher score)

**Table 3:** Ten main solutions to patient safety threats in homecare

RANK	Proposed solutions to homecare safety threats	Total Priority Score	Types of contributing factors to safety and quality in home health care	Type of activity
1.	Encourage family members to participate in care and offer them free training	97.7	Carers	Education & Family Involvement
2.	Carers to receive training on the use of equipment, safe patient transfers and how to physically support the patients so that they do not hurt themselves or the patient	97.4	Carers	Education
3.	Carry out reviews for family members acting as carers to ensure that they are coping	96.4	Clinicians & Carers	Review and supervision
4.	To have home visits from a community dementia nurse in order to identify those at risk, the triggers and signs and any changes in the condition	96.1	Clinicians & Carers	Review and supervision
5.	Carers to attend regular training on all aspects of dementia care and management of certain behaviours	95.4	Carers	Education
6.	Train carers in the basics of giving medication and vital signs check	94.4	Carers	Education
7.	Offer special training in dementia for GPs	94.1	Clinicians	Organization of care & Education
8.	Encourage relatives or carers to attend appointments with the patient	94.1	Clinicians	Family Involvement
9.	Make adjustments and provide safe care in the home environment	93.8	Home environment	Safety proofing
10.	Carers to have regular supervision by a senior person to support them and to identify any additional training requirements	93.5	Carers	Review and supervision

(Clinicians scored problems using the following criteria: frequency, severity, inequity, economic impact and responsiveness to solution (Table 1). The scoring options were 1 for “yes (e.g. this problem is common)”, 0 for “no (e.g. this problem is uncommon)”, 0.5 for “unsure (e.g. I am unsure if this problem is common)” and blank for “unaware e.g. I do not know if his problem is common)”. Total Priority score is the mean of the scores for each of the five criteria and is ranging from 0 to 100. Higher ranked problems received more “Yes” responses for each of the criteria and a higher score)

The highest ranked problems relating to homecare safety in people with dementia focused mostly on carers and patients. The top carer-related problems focused on a need for education, qualification and training, carers’ inability to cope, deterioration of their health and burnout. Main patient-related problems focused on patients neglecting themselves, experiencing social isolation, having poor mobility, forgetting to take medications and not knowing how or when to seek help. Lower socio-economic groups or ethnic minorities were considered more likely to be affected by reduced GP budgets, day centres and social care/services resources, to have carers with inappropriate education and training, to be socially isolated and to have unsafe environment.

Overall, the proposed problems in homecare safety of patients with dementia mainly addressed carer-related issues (Additional file 1: Appendix 4). In most cases, these suggestions either referred to both family and formal carers or this was not clearly specified. Carer-related suggestions, included in the top 10 priorities, mainly addressed carers’ condition and health. The lower ranked suggestions focused on the issues in carer-person with dementia relationship such as poor communication, neglect and lack of support and sensitivity.

Overall, the identified solutions mostly focused on carers (Additional file 1: Appendix 5). A number of the proposed solutions identified a need for improving professional carers’ recruitment, supervision, education and working conditions. Several solutions focused on clinicians’ role in carers’ supervision and organisation of care and home environment safety proofing using e.g. alarmed doors, safety buzzers, dementia friendly ovens or locks.

The comparison between problems and solutions showed some correlation as both groups of suggestions emphasised the role of carers. While several highly ranked problems focused on people with dementia, solutions were mostly aimed at carers, indicating that carers are seen as the key answer to many patient-related homecare threats. Education of all homecare-related stakeholders was underscored as a suitable response to a number of proposed safety threats. The highest ranked suggestions had the highest AEA, i.e. there was a stronger agreement among the clinicians in regards to the top suggestions compared to those ranked lower which had a significant number of “Unsure” and “Unaware” answers to scoring.

## Discussion

In this study, dementia care clinicians identified 27 homecare safety problems and 30 solutions for dementia patients. The collated suggestions covered a range of interventions relating to carers, patients, clinicians, home environment, organization and provision of care. The top ranked homecare safety problems focused on inadequate education of both family and professional carers and challenges faced by patients (e.g. self-neglect, social isolation, medication nonadherence etc.). The top ranked solutions focused on involvement, training and education of family carers as well as supervision and continuing support to ensure they are coping. Identified priorities also highlighted a need for improving recruitment, oversight and working conditions of professional carers and included different strategies for home safety-proofing.

Carers are the key actors in ensuring homecare safety of people with dementia as confirmed across both the proposed problems and solutions. A number of suggestions in this study relate to the importance of carers’ health and wellbeing. This corresponds to the literature showing that dementia care tends to be longer, more demanding and detrimental to carers compared to other types of caregiving [21]. Most of the proposed solutions shifted the responsibility for provision of safe dementia care from healthcare services to families while focussing on carers’ education, supervision and support. Multicomponent interventions aimed at carers, comprising training, aid, guidance and respite, have been shown to maintain their mood and morale, reduce strain and reduce or delay transition from home into a care home [7, 22, 23]. Presently, their uptake is minimal as no government can afford scaling-up provision of these interventions throughout the dementia care system [7]. However, if direct care for people with dementia is unfeasible, it is essential to provide carers with access to a range of support such as financial, emotional and physical assistance.

The most important threats to homecare safety identified by the clinicians were reduced GP budgets as well as day centres and social services resources. GP budgets in the UK context refer to the budgets available to the Clinical commissioning groups (CCGs). CCGs consist of local GP practices as members and are led by an elected Governing Body largely made of GPs. As one of the statutory NHS bodies, CCGs are responsible for the planning and commissioning of healthcare services for their local area, including mental health services, urgent and emergency care, elective hospital services, and community care [24]. A recent analysis shows that spending on care for people aged 65 and over has fallen by a fifth in England over the last 10 years [25]. A survey of carers of people with dementia in the UK showed that fewer than 20% thought they received enough support from the government [26]. The need for larger financial support from their governments is noted by carers throughout Europe [27].

The identified solutions correspond to the actions proposed in the UK government's five year vision for the future of dementia care launched in 2015 such as provision of meaningful and supportive care to patients and families, raising public awareness, ensuring equal and quick access to diagnosis, counting on GPs coordination and continuity of care, training all NHS staff on dementia, reducing inappropriate prescribing of antipsychotic medication and improving professional caregivers’ working conditions [28].

### Strengths and limitations

In this study, we used a modified version of a widely-adopted research priority-setting methodology. In previous surveys, the main causes and solutions to patient safety were identified in terms of how frequently they occurred [29, 30]. Our study uses a broader set of criteria satisfying all the three main dimensions of public health benefit (should we do it?), feasibility (can we do it?) and cost [31]. PRIORITIZE is founded on a notion of harnessing collective wisdom for better decision-making, recognised as one of the key challenges for social science [32]. This crowdsourcing approach is particularly useful to improve our understanding of topics that are emotionally laden, charged with guilt or risk of blame and preferably avoided such as patient safety [33].

Physicians are often unwilling to participate in surveys and the low response rate in this study corresponds to other clinicians’ surveys [34, 35]. Longer, online surveys and those with open-ended questions (such as our survey) are particularly prone to poor response rate [36, 37]. Embedding this approach into the organizational quality improvement process in a longitudinal manner could lead to increased ownership, better response rate and richer patient safety-related information. Another limitation of this study concerns generalizability and validity of the findings. The respondents were self-selected and potentially differed from the non-respondents, e.g. by being more motivated and better informed than the non-responders and perhaps choosing different priorities. We believe this is unlikely as all invited participants share the same eligibility criteria as clinicians providing dementia care in North-West London; there may have however been other biases that were not measured. Furthermore, collated clinicians’ suggestions often referred to both family and formal carers or this was not clearly specified.

The PRIORITIZE approach is at an early stage and could benefit from further refinement. For example, provision of examples to guide the specificity and type of the suggestions (e.g. error producing conditions, errors and adverse events), adding a longitudinal perspective through repeated annual surveys or including different types of participants (e.g. patients or carers) could be beneficial. This approach also offers possibility of different types of analysis, e.g. determining the level of the intervention implementation, choosing different prioritization criteria, evaluating the highest ranked suggestions according to individual scoring criteria or undertaking an in-depth comparison of clinicians’ and patients’ views.

## Conclusions

The demands of dementia homecare call for inclusion of all relevant stakeholders in the development, implementation and evaluation of robust quality and safety initiatives. Clinicians, as the providers and custodians of quality in dementia care, have a vital say on priorities for homecare safety of people with dementia. In our study, clinicians identified some challenging and costly suggestions but also a range of affordable and feasible suggestions for improvement of homecare safety of people with dementia. The variety of identified priorities uncovered a need for integration and collaboration of different dementia care providers, such as carers, family members, patients, clinicians, homecare organizations and policy-makers, to ensure safety of dementia patients at home. Some suggestions were synergistic or inter-related (e.g. “Professional carers lacking proper training and qualifications”, “Carers to receive training on the use of equipment, safe patient transfers and how to physically support the patients so that they do not hurt themselves or the patient”, “Train carers in the basics of giving medication and vital signs check”), reaffirming the importance of certain themes and conveying a clear message where action is needed.

This approach is in alignment with recent policy decisions to involve healthcare staff in patient safety research [38]. Our findings open an opportunity to add to the limited research literature on patient safety in dementia homecare by evaluating the congruence between the proposed priorities, currently implemented policies and available research evidence. The priority setting approach could be introduced into healthcare and social care quality control as part of a quality improvement initiative to detect the vulnerabilities at different stages, levels, and dimensions of dementia care.

## Additional file

Additional file 1: Appendix 1. Initial questionnaire on problems and solutions related to homecare safety of people with dementia. Appendix 2. Scoring questionnaire. Appendix 3. Characteristics of the respondents to the initial questionnaire. Appendix 4. Ranking of all (30) home care safety-related problems from clinicians’ perspective (AEA: 0 to 1). Appendix 5. Ranking of all (31) solutions to home care safety threats from clinicians’ perspective (AEA: 0 to 1). (DOCX 121 kb)

## Abbreviations

AEA: Average experts’ agreement
GP: General practitioners
