# Supplementary material for: Prioritizing problems in and solutions to homecare safety of people with dementia: supporting carers, streamlining care
Source: BMC Geriatr. 2017 Jan 19;17:26. doi: 10.1186/s12877-017-0415-6 (PMC5244548; doi:10.1186/s12877-017-0415-6)
Supplement: Additional file 1: — Appendix 1. Initial questionnaire on problems and solutions related to homecare safety of people with dementia. Appendix 2. Scoring questionnaire. Appendix 3. Characteristics of the respondents to the initial questionnaire. Appendix 4. Ranking of all (30) home care safety-related problems from clinicians’ perspective (AEA: 0 to 1). Appendix 5. Ranking of all (31) solutions to home care safety threats from clinicians’ perspective (AEA: 0 to 1). (DOCX 121 kb) [file 12877_2017_415_MOESM1_ESM.docx]

# Additional File 1

**Appendix 1. Initial questionnaire on problems and solutions related to homecare safety of people with dementia**

| 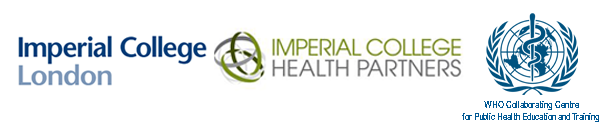 | | | | | | | |
| --- | --- | --- | --- | --- | --- | --- | --- |
| **Patient safety in dementia care**      Dear *Colleague*,    This survey aims to capture (1) *your* perspective and (2) *your* solutions for incidents that affect the safety of patients with dementia. It has been designed by the Department of Primary Care and Public Health, Imperial College and Imperial College Health Partners to guide patient safety improvement initiatives in NW London and on a wider scale. The results will be used anonymously. | | | | | | | |
|  | | | | | | | |
| Please specify your role: | | | | | | | |
| Nurse | | Specialist Trainee | Consultant | GP | Social Care Professional | Pharmacist | Other (please specify)  _________________ |
| ○ | | ○ | ○ | ○ | ○ | ○ | ○ |
|  | | | | | | | |
|  | | | | | | | |
| **1. Medication-related problems**  A. Please name 3 main medication-related problems affecting the safety of patients with dementia. | | | | | | | |
|  |  | | | | | | |
|  | | | | | | | |
| B. How could we reduce or prevent medication-related problems affecting the safety of patients with dementia? | | | | | | | |
|  |  | | | | | | |
|  | | | | | | | |
| **2. Home care-related problems**  A. Please name 3 main home care-related problems affecting the safety of patients with dementia. | | | | | | | |
|  |  | | | | | | |
|  | | | | | | | |
| B. How could we reduce or prevent home care-related problems affecting the safety of patients with dementia? | | | | | | | |
|  |  | | | | | | |
|  | | | | | | | |
| **3. Keeping an eye on care**  How could patient safety be better monitored in a GP surgery or a hospital? | | | | | | | |
|  |  | | | | | | |
|  | | | | | | | |
| **4. What's on your mind?**  Kindly give any other suggestions for how the care of people with dementia could be made safer? | | | | | | | |
|  |  | | | | | | |
|  | | | | | | | |
|  | | | | | | | |
| **Thank you very much for your time and effort.**  Your contribution will help improve patient safety in dementia care. For more information and to learn more about the outcomes of the study, please contact Dr. Josip Car at [josip.car@imperial.ac.uk](mailto:josip.car@imperial.ac.uk) | | | | | | | |

**Appendix 2. Scoring questionnaire**


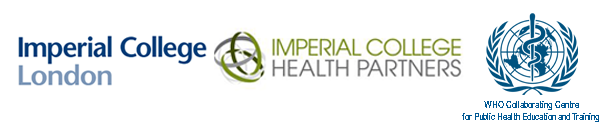


|  | | | | | | | | | | | | | | | | | | | | | |  |  |  |  |  |  |  |  |  |  |
| --- | --- | --- | --- | --- | --- | --- | --- | --- | --- | --- | --- | --- | --- | --- | --- | --- | --- | --- | --- | --- | --- | --- | --- | --- | --- | --- | --- | --- | --- | --- | --- |
| **Patient safety priorities in dementia care**    Dear *Colleague,*    Thank you for participating in the first step of this process where you identified patient safety problems in home care for people with dementia and proposed solutions. They are all listed below. In this second and final step, please indicate your agreement or disagreement with all the statements in the table below using the following criteria:  **Y(es) : if you agree**  **N(o) : if you disagree**  **UnS(ure) : if you are aware of the problem, but unsure about the answer**  **UnA(ware) : if you are not sufficiently aware of the problem** | | | | | | | | | | | | | | | | | | | | | |  |  |  |  |  |  |  |  |  |  |
|  | | | | | | | | | | | | | | | | | | | | | |  |  |  |  |  |  |  |  |  |  |
|  | Y | N | UnS | UnA | | Y | | N | | UnS | | UnA | Y | N | UnS | | UnA | | Y | N | UnS | UnA | Y | | | | N | UnS | |  |  |
| 01. Carers burnout | ○ | ○ | ○ | ○ | | ○ | | ○ | | ○ | | ○ | ○ | ○ | ○ | | ○ | | ○ | ○ | ○ | ○ | ○ | | | | ○ | ○ | | ○ |  |
| 02. Health deterioration in family members due to burden of caring | ○ | ○ | ○ | ○ | | ○ | | ○ | | ○ | | ○ | ○ | ○ | ○ | | ○ | | ○ | ○ | ○ | ○ | ○ | | | | ○ | ○ | | ○ |  |
| 03. Lack of continuity of care | ○ | ○ | ○ | ○ | | ○ | | ○ | | ○ | | ○ | ○ | ○ | ○ | | ○ | | ○ | ○ | ○ | ○ | ○ | | | | ○ | ○ | | ○ |  |
| 04. Lack of flexibility of care packages | ○ | ○ | ○ | ○ | | ○ | | ○ | | ○ | | ○ | ○ | ○ | ○ | | ○ | | ○ | ○ | ○ | ○ | ○ | | | | ○ | ○ | | ○ |  |
| 07. Carers lacking training and education | ○ | ○ | ○ | ○ | | ○ | | ○ | | ○ | | ○ | ○ | ○ | ○ | | ○ | | ○ | ○ | ○ | ○ | ○ | | | | ○ | ○ | | ○ |  |
| 09. Carers rushing through care | ○ | ○ | ○ | ○ | | ○ | | ○ | | ○ | | ○ | ○ | ○ | ○ | | ○ | | ○ | ○ | ○ | ○ | ○ | | | | ○ | ○ | | ○ |  |
| 10. Carers refusing to give medication to the patient | ○ | ○ | ○ | ○ | | ○ | | ○ | | ○ | | ○ | ○ | ○ | ○ | | ○ | | ○ | ○ | ○ | ○ | ○ | | | | ○ | ○ | | ○ |  |
| 11. Carers lacking proper training and qualifications | ○ | ○ | ○ | ○ | | ○ | | ○ | | ○ | | ○ | ○ | ○ | ○ | | ○ | | ○ | ○ | ○ | ○ | ○ | | | | ○ | ○ | | ○ |  |
| 12. Carers lacking sensitivity and respect for the patient | ○ | ○ | ○ | ○ | | ○ | | ○ | | ○ | | ○ | ○ | ○ | ○ | | ○ | | ○ | ○ | ○ | ○ | ○ | | | | ○ | ○ | | ○ |  |
| 13. Family members unable to manage the patient | ○ | ○ | ○ | ○ | | ○ | | ○ | | ○ | | ○ | ○ | ○ | ○ | | ○ | | ○ | ○ | ○ | ○ | ○ | | | | ○ | ○ | | ○ |  |
| 14. Carers unaware or not knowing how to use equipment or adaptations to reduce patients risk of health hazards | ○ | ○ | ○ | ○ | | ○ | | ○ | | ○ | | ○ | ○ | ○ | ○ | | ○ | | ○ | ○ | ○ | ○ | ○ | | | | ○ | ○ | | ○ |  |
| 15. Lack of family support, contribution and involvement in care | ○ | ○ | ○ | ○ | | ○ | | ○ | | ○ | | ○ | ○ | ○ | ○ | | ○ | | ○ | ○ | ○ | ○ | ○ | | | | ○ | ○ | | ○ |  |
| 16. Poor mobility and falls | ○ | ○ | ○ | ○ | | ○ | | ○ | | ○ | | ○ | ○ | ○ | ○ | | ○ | | ○ | ○ | ○ | ○ | ○ | | | | ○ | ○ | | ○ |  |
| 17. Patients unable to express themselves, falsely creating an impression of coping | ○ | ○ | ○ | ○ | | ○ | | ○ | | ○ | | ○ | ○ | ○ | ○ | | ○ | | ○ | ○ | ○ | ○ | ○ | | | | ○ | ○ | | ○ |  |
| 18. No safety alarm/ button in patient’s surrounding | ○ | ○ | ○ | ○ | | ○ | | ○ | | ○ | | ○ | ○ | ○ | ○ | | ○ | | ○ | ○ | ○ | ○ | ○ | | | | ○ | ○ | | ○ |  |
| 19. Unfamiliar carers causing anxiety and agitation in patients | ○ | ○ | ○ | ○ | | ○ | | ○ | | ○ | | ○ | ○ | ○ | ○ | | ○ | | ○ | ○ | ○ | ○ | ○ | | | | ○ | ○ | | ○ |  |
| 20. Unsafe design of home environment | ○ | ○ | ○ | ○ | | ○ | | ○ | | ○ | | ○ | ○ | ○ | ○ | | ○ | | ○ | ○ | ○ | ○ | ○ | | | | ○ | ○ | | ○ |  |
| 21. Patient left unattended | ○ | ○ | ○ | ○ | | ○ | | ○ | | ○ | | ○ | ○ | ○ | ○ | | ○ | | ○ | ○ | ○ | ○ | ○ | | | | ○ | ○ | | ○ |  |
| 22. Patient forgetting where or how to seek help when needed | ○ | ○ | ○ | ○ | | ○ | | ○ | | ○ | | ○ | ○ | ○ | ○ | | ○ | | ○ | ○ | ○ | ○ | ○ | | | | ○ | ○ | | ○ |  |
| 23. Patient forgetting to take the medication | ○ | ○ | ○ | ○ | | ○ | | ○ | | ○ | | ○ | ○ | ○ | ○ | | ○ | | ○ | ○ | ○ | ○ | ○ | | | | ○ | ○ | | ○ |  |
| 24. Patient neglecting themselves | ○ | ○ | ○ | ○ | | ○ | | ○ | | ○ | | ○ | ○ | ○ | ○ | | ○ | | ○ | ○ | ○ | ○ | ○ | | | | ○ | ○ | | ○ |  |
| 25. Social Isolation | ○ | ○ | ○ | ○ | | ○ | | ○ | | ○ | | ○ | ○ | ○ | ○ | | ○ | | ○ | ○ | ○ | ○ | ○ | | | | ○ | ○ | | ○ |  |
| 26. Suboptimal prevention and treatment of pressure sores | ○ | ○ | ○ | ○ | | ○ | | ○ | | ○ | | ○ | ○ | ○ | ○ | | ○ | | ○ | ○ | ○ | ○ | ○ | | | | ○ | ○ | | ○ |  |
| 27. Lack of adherence to a care regime | ○ | ○ | ○ | ○ | | ○ | | ○ | | ○ | | ○ | ○ | ○ | ○ | | ○ | | ○ | ○ | ○ | ○ | ○ | | | | ○ | ○ | | ○ |  |
| 28. Lack of a regular and familiar routine | ○ | ○ | ○ | ○ | | ○ | | ○ | | ○ | | ○ | ○ | ○ | ○ | | ○ | | ○ | ○ | ○ | ○ | ○ | | | | ○ | ○ | | ○ |  |
| 29. Reduced GP budgets, day centers and social care/services resources | ○ | ○ | ○ | ○ | | ○ | | ○ | | ○ | | ○ | ○ | ○ | ○ | | ○ | | ○ | ○ | ○ | ○ | ○ | | | | ○ | ○ | | ○ |  |
| 30. Difficulty for health professionals to obtain a clear history | ○ | ○ | ○ | ○ | | ○ | | ○ | | ○ | | ○ | ○ | ○ | ○ | | ○ | | ○ | ○ | ○ | ○ | ○ | | | | ○ | ○ | | ○ |  |
| **Please add any further ideas or comments that you believe are important and should be included in this study.** | | | | | | | | | | | | | | | | | | | | | | | | | |  |  |  |  |  |  |
|  |  | | | | | | | | | | | | | | | | | | | | | | | | | | | | | | |
| **Y(es) : if you agree**  **N(o) : if you disagree**  **UnS(ure) : if you are aware of the problem, but unsure about the answer**  **UnA(ware) : if you are not sufficiently aware of the problem** | | | | | | | | | | | | | | | | | | | | | | | | | |  |  |  |  |  |  |
| **Criteria for ranking of solutions to homecare safety threats in people with dementia** | | | | | This solution is cost-effective | | | | | | | | | | | The implementation of this solution is feasible | | | | | | | | | This solution would save lives | | | | | | |
|  | | | | | Y | | N | | UnS | | UnA | | | | | Y | | N | UnS | | | UnA | | Y | | | N | UnS | UnA | | |
| 01. Establish continuity and consistency of care with as few carers as possible | | | | | ○ | | ○ | | ○ | | ○ | | | | | ○ | | ○ | ○ | | | ○ | | ○ | | | ○ | ○ | ○ | | |
| 02. If continuity of care is not an option, strongly encourage the patient to move into a residential home | | | | | ○ | | ○ | | ○ | | ○ | | | | | ○ | | ○ | ○ | | | ○ | | ○ | | | ○ | ○ | ○ | | |
| 03. Encourage family members to participate in care and offer them free training | | | | | ○ | | ○ | | ○ | | ○ | | | | | ○ | | ○ | ○ | | | ○ | | ○ | | | ○ | ○ | ○ | | |
| 04. Make carers registration and vetting more rigorous | | | | | ○ | | ○ | | ○ | | ○ | | | | | ○ | | ○ | ○ | | | ○ | | ○ | | | ○ | ○ | ○ | | |
| 05. Care agencies to use mobile tracking devices to monitor their staff | | | | | ○ | | ○ | | ○ | | ○ | | | | | ○ | | ○ | ○ | | | ○ | | ○ | | | ○ | ○ | ○ | | |
| 06. Remuneration of carers needs to be such as to attract quality candidates with the right values and allow them to also have career progression | | | | | ○ | | ○ | | ○ | | ○ | | | | | ○ | | ○ | ○ | | | ○ | | ○ | | | ○ | ○ | ○ | | |
| 07. Dismiss carers who do not have the correct values despite the training | | | | | ○ | | ○ | | ○ | | ○ | | | | | ○ | | ○ | ○ | | | ○ | | ○ | | | ○ | ○ | ○ | | |
| 08. Agencies to match the patient with a carer and ensure they meet patient’s needs at all times as feasibly possible | | | | | ○ | | ○ | | ○ | | ○ | | | | | ○ | | ○ | ○ | | | ○ | | ○ | | | ○ | ○ | ○ | | |
| 09. If change of carer needs to occur, the new carer is introduced slowly | | | | | ○ | | ○ | | ○ | | ○ | | | | | ○ | | ○ | ○ | | | ○ | | ○ | | | ○ | ○ | ○ | | |
| 10. Aim for two carers per patient to ensure consistency of care | | | | | ○ | | ○ | | ○ | | ○ | | | | | ○ | | ○ | ○ | | | ○ | | ○ | | | ○ | ○ | ○ | | |
| 11. Try to provide option for familiar family faces to stay in the hospital with the patient to decrease the patient’s deterioration and disorientation | | | | | ○ | | ○ | | ○ | | ○ | | | | | ○ | | ○ | ○ | | | ○ | | ○ | | | ○ | ○ | ○ | | |
| 12. Encourage relatives or carers to attend appointments with the patient | | | | | ○ | | ○ | | ○ | | ○ | | | | | ○ | | ○ | ○ | | | ○ | | ○ | | | ○ | ○ | ○ | | |
| 13. Carers to attend regular training on all aspects of dementia care and management of certain behaviours | | | | | ○ | | ○ | | ○ | | ○ | | | | | ○ | | ○ | ○ | | | ○ | | ○ | | | ○ | ○ | ○ | | |
| 14. Carers to receive training on the use of equipment, safe patient transfers and how to physically support the patients so that they do not hurt themselves or the patient | | | | | ○ | | ○ | | ○ | | ○ | | | | | ○ | | ○ | ○ | | | ○ | | ○ | | | ○ | ○ | ○ | | |
| 15. Train carers in the basics of giving medication and vital signs check | | | | | ○ | | ○ | | ○ | | ○ | | | | | ○ | | ○ | ○ | | | ○ | | ○ | | | ○ | ○ | ○ | | |
| 16. Carers to have regular supervision by a senior person to support them and to identify any additional training requirements | | | | | ○ | | ○ | | ○ | | ○ | | | | | ○ | | ○ | ○ | | | ○ | | ○ | | | ○ | ○ | ○ | | |
| 17. Create an expert advice line for informal carers which they can consult 24/7 | | | | | ○ | | ○ | | ○ | | ○ | | | | | ○ | | ○ | ○ | | | ○ | | ○ | | | ○ | ○ | ○ | | |
| 18. Provide respite for carers and relatives | | | | | ○ | | ○ | | ○ | | ○ | | | | | ○ | | ○ | ○ | | | ○ | | ○ | | | ○ | ○ | ○ | | |
| 19. Increased publicity about the problems of dementia patients | | | | | ○ | | ○ | | ○ | | ○ | | | | | ○ | | ○ | ○ | | | ○ | | ○ | | | ○ | ○ | ○ | | |
| 21. Develop a “zero harm” culture | | | | | ○ | | ○ | | ○ | | ○ | | | | | ○ | | ○ | ○ | | | ○ | | ○ | | | ○ | ○ | ○ | | |
| 22. Transport services to phone ahead of time to let patients know that they will be arriving to pick them up | | | | | ○ | | ○ | | ○ | | ○ | | | | | ○ | | ○ | ○ | | | ○ | | ○ | | | ○ | ○ | ○ | | |
| 23. Make adjustments and provide safe care in the home environment | | | | | ○ | | ○ | | ○ | | ○ | | | | | ○ | | ○ | ○ | | | ○ | | ○ | | | ○ | ○ | ○ | | |
| 24. Install alarmed doors and safety buzzers | | | | | ○ | | ○ | | ○ | | ○ | | | | | ○ | | ○ | ○ | | | ○ | | ○ | | | ○ | ○ | ○ | | |
| 25. Increase the patient’s familiarity with the environment | | | | | ○ | | ○ | | ○ | | ○ | | | | | ○ | | ○ | ○ | | | ○ | | ○ | | | ○ | ○ | ○ | | |
| 26. Provide dementia friendly locks | | | | | ○ | | ○ | | ○ | | ○ | | | | | ○ | | ○ | ○ | | | ○ | | ○ | | | ○ | ○ | ○ | | |
| 27. Provide a dementia friendly oven | | | | | ○ | | ○ | | ○ | | ○ | | | | | ○ | | ○ | ○ | | | ○ | | ○ | | | ○ | ○ | ○ | | |
| 28. Offer special training in dementia for GPs | | | | | ○ | | ○ | | ○ | | ○ | | | | | ○ | | ○ | ○ | | | ○ | | ○ | | | ○ | ○ | ○ | | |
| 29. Carry out reviews for family members acting as carers to ensure that they are coping | | | | | ○ | | ○ | | ○ | | ○ | | | | | ○ | | ○ | ○ | | | ○ | | ○ | | | ○ | ○ | ○ | | |
| 30. The carer to have a “simple timetable care plan” for each identified problem and to try to maintain a simple daily routine | | | | | ○ | | ○ | | ○ | | ○ | | | | | ○ | | ○ | ○ | | | ○ | | ○ | | | ○ | ○ | ○ | | |
| 31. To have home visits from a community dementia nurse in order to identify those at risk, the triggers and signs and any changes in the condition | | | | | ○ | | ○ | | ○ | | ○ | | | | | ○ | | ○ | ○ | | | ○ | | ○ | | | ○ | ○ | ○ | | |
| **Please add any further ideas or comments that you believe are important and should be included in this study.** | | | | | | | | | | | | | | | | | | | | | | | | | | | | | | | |
| **Please add any further ideas or comments that you believe are important and should be included in this study.** | | | | | | | | | | | | | | | | | | | | | | | | | | | | | | | |
| **Is there anything else for patient safety in home care for people with dementia that you would like to share with us?** | | | | | | | | | | | | | | | | | | | | | | | | | | | | | | | |
| **Thank you very much for your time and effort.**  Your contribution will help improve patient safety in dementia care.  For more information and to learn more about the outcomes of the study, please contact Dr. Josip Car at [josip.car@imperial.ac.uk](mailto:josip.car@imperial.ac.uk) | | | | | | | | | | | | | | | | | | | | | | | | | | | | | | | |

**Appendix 3. Characteristics of the respondents**

Total number of survey respondents: 76

- GPs: 34, 44.7%
- GP Trainees: 7, 9.2%
- Nurse: 15, 19.7%
- Psychologist: 3, 3.9%
- Social Care Professional: 3, 3.9%
- Occupational Therapist: 3, 3.9%
- Blank: 3, 3.9%
- Pharmacists: 2, 2.6%
- Nursing assistant: 1, 1.3%
- Allied Health Professional: 1, 1.3%
- Trust SHO: 1, 1.3%
- Consultants: 1, 1.3%
- Dementia befriending Coordinator: 1, 1.3%
- Speech and language therapist: 1, 1.3%

**Appendix 4. Ranking of all (30) home care safety-related problems from clinicians’ perspective (AEA: 0 to 1)**

| **Highlighted home care-related problems in dementia care** | **Type of the actor or setting related to home care safety problems** | **Type of the contributory factor leading to home care safety problems** | **TPS** | **AEA** | **Responsiveness to solution** | **Frequency** | **Economic impact** | **Severity** | **Inequity** |
| --- | --- | --- | --- | --- | --- | --- | --- | --- | --- |
| *Reduced GP budgets, day centres and social care/services resources* | System & organizational | Resources | 1 | **0,75** | 17 | 5 | 4 | 4 | 1 |
| *Professional carers lacking proper training and qualifications* | Clinicians | Knowledge and skills | 2 | **0,73** | 3 | 12 | 11 | 9 | 2 |
| *Family carers lacking training and education* | Carers | Knowledge and skills | 3 | **0,71** | 1 | 14 | 13 | 5 | 5 |
| *Patient neglecting themselves* | Patient | Support | 4 | **0,73** | 24 | 2 | 2 | 3 | 13 |
| *Social Isolation* | Patient & Carers | Support; Patient-carer relationship | 5 | **0,70** | 20 | 4 | 17 | 10 | 3 |
| *Patient forgetting to take the medication* | Patient | Support | 6 | **0,72** | 19 | 1 | 3 | 2 | 28 |
| *Unsafe design of home environment* | Home environment | Safe setting | 7 | **0,69** | 16 | 11 | 14 | 13 | 4 |
| *Poor mobility and falls* | Patient | Support | 8 | **0,75** | 21 | 3 | 1 | 1 | 30 |
| *Family members unable to manage the patient* | Carers | Knowledge and skills; Support | 9 | **0,68** | 18 | 8 | 6 | 7 | 20 |
| *Health deterioration in family members due to burden of caring* | Carers | Support | 10 | **0,65** | 22 | 6 | 7 | 22 | 7 |
| *Patient forgetting where or how to seek help when needed* | Patient | Support | 11 | **0,69** | 29 | 10 | 9 | 6 | 16 |
| *Suboptimal prevention and treatment of pressure sores* | Clinicians | Provision of care | 12 | **0,62** | 10 | 24 | 10 | 12 | 17 |
| *Carers’ burnout* | Carers | Support | 13 | **0,65** | 25 | 7 | 5 | 30 | 6 |
| *Lack of continuity of care* | System and organizational | Organization of care | 14 | **0,64** | 7 | 17 | 12 | 14 | 25 |
| *Patient left unattended* | Carers | Provision of care | 15 | **0,64** | 26 | 18 | 8 | 8 | 15 |
| *Unfamiliar carers causing anxiety and agitation in patients* | Carers | Organisation of care; Patient-carer relationship | 16 | **0,62** | 8 | 9 | 27 | 21 | 24 |
| *Carers unaware or not knowing how to use equipment or adaptations to reduce patients risk of health hazards* | Carers | Knowledge and skills | 17 | **0,64** | 4 | 20 | 22 | 16 | 12 |
| *Lack of adherence to a care regime* | Carers | Provision of care | 18 | **0,62** | 12 | 19 | 15 | 15 | 19 |
| *Carers rushing through care* | Carers | Provision of care | 19 | **0,59** | 6 | 15 | 29 | 20 | 18 |
| *Patients unable to express themselves, falsely creating an impression of coping* | Patient | Support | 20 | **0,66** | 23 | 13 | 18 | 11 | 26 |
| *Lack of flexibility of care packages* | System & organizational | Provision of care | 21 | **0,61** | 5 | 23 | 24 | 25 | 9 |
| *No safety alarm/ button in patient’s surrounding* | Home environment | Setting | 22 | **0,61** | 2 | 27 | 19 | 28 | 23 |
| *Lack of a regular and familiar routine* | Carers | Provision of care | 23 | **0,58** | 14 | 16 | 23 | 29 | 14 |
| *Poor relationship and communication between the patient and the carer* | Carers & Patient | Patient-carer relationship | 24 | **0,58** | 15 | 25 | 28 | 19 | 10 |
| *Carers lacking sensitivity and respect for the patient* | Carers | Patient-carer relationship; Provision of care | 25 | **0,56** | 11 | 21 | 30 | 26 | 11 |
| *Difficulty for health professionals to obtain a clear history* | Clinicians & Patient | Provision of care | 26 | **0,60** | 30 | 26 | 20 | 27 | 8 |
| *Lack of family support, contribution and involvement in care* | Carers | Patient-carer relationship | 27 | **0,62** | 27 | 22 | 16 | 17 | 27 |
| *Patient neglect by carer, e.g. not given sufficient food or drink* | Carers | Provision of care | 28 | **0,54** | 13 | 28 | 25 | 18 | 21 |
| *Carers refusing to give medication to the patient* | Carers | Provision of care | 29 | **0,55** | 9 | 29 | 21 | 24 | 29 |
| *Physical abuse of patient by the carers or relatives* | Carers | Patient-carer relationship | 30 | **0,51** | 28 | 30 | 26 | 23 | 22 |

***AEA –average expert agreement; TPS – total priority score***

**Appendix 5. Ranking of all (31) solutions to home care safety threats from clinicians’ perspective (AEA: 0 to 1)**

| **Proposed solution for threats to home-care safety of people with dementia** | **Type of the actor or setting addressed by the proposed home care safety solution** | **Type of the activity proposed for improvement of home care safety** | **TPS** | **AEA** | **Cost-effectiveness** | **Feasibility** | **Saving Lives** |
| --- | --- | --- | --- | --- | --- | --- | --- |
| *Encourage family members to participate in care and offer them free training* | Carers | Education; Family involvement | **1** | **0,95** | **1** | **1** | **3** |
| *Carers to receive training on the use of equipment, safe patient transfers and how to physically support the patients so that they do not hurt themselves or the patient* | Carers | Education | **2** | **0,96** | **4** | **2** | **1** |
| *Carry out reviews for family members acting as carers to ensure that they are coping* | Clinicians & Carers | Review and supervision | **3** | **0,93** | **2** | **3** | **11** |
| *To have home visits from a community dementia nurse in order to identify those at risk, the triggers and signs and any changes in the condition* | Clinicians & Carers | Review and supervision | **4** | **0,93** | **6** | **8** | **5** |
| *Carers to attend regular training on all aspects of dementia care and management of certain behaviours* | Carers | Education | **5** | **0,93** | **9** | **9** | **2** |
| *Train carers in the basics of giving medication and vital signs check* | System & Carers | Education | **6** | **0,89** | **5** | **5** | **12** |
| *Offer special training in dementia for GPs* | Clinicians | Education | **7** | **0,91** | **7** | **12** | **7** |
| *Encourage relatives or carers to attend appointments with the patient* | Clinicians | Family involvement | **8** | **0,90** | **3** | **4** | **14** |
| *Make adjustments and provide safe care in the home environment* | Home environment | Safety proofing | **9** | **0,90** | **14** | **10** | **6** |
| *Carers to have regular supervision by a senior person to support them and to identify any additional training requirements* | Carers | Review and supervision | **10** | **0,90** | **12** | **13** | **4** |
| *Increased publicity about the problems of dementia patients* | Public | Advocacy | **11** | **0,86** | **13** | **6** | **16** |
| *Create an expert advice line for informal carers which they can consult 24/7* | Carers | Organization of care | **12** | **0,86** | **17** | **17** | **8** |
| *Increase the patient’s familiarity with the environment* | Patient | Organization of care | **13** | **0,82** | **10** | **14** | **21** |
| *The carer to have a “simple timetable care plan” for each identified problem and to try to maintain a simple daily routine* | Carers | Organization of care | **14** | **0,83** | **11** | **15** | **23** |
| *Provide respite for carers and relatives* | Carers | Support | **15** | **0,86** | **21** | **11** | **9** |
| *Care packages need to be more flexible* | Carers & Patient | Organization of care | **16** | **0,79** | **18** | **18** | **15** |
| *Install alarmed doors and safety buzzers* | Home environment | Safety proofing | **17** | **0,83** | **22** | **19** | **10** |
| *Remuneration of carers needs to be such as to attract quality candidates with the right values and allow them to also have career progression* | Carers | Working conditions | **18** | **0,78** | **24** | **16** | **13** |
| *Make carers registration and vetting more rigorous* | Carers | Recruitment and vetting | **19** | **0,82** | **19** | **20** | **22** |
| *Provide dementia friendly locks* | Home environment | Safety proofing | **20** | **0,78** | **23** | **22** | **19** |
| *Establish continuity and consistency of care with as few carers as possible* | Carers | Organization of care | **21** | **0,79** | **8** | **27** | **25** |
| *Agencies to match the patient with a carer and ensure they meet patient’s needs at all times as feasibly possible* | Carers | Organization of care | **22** | **0,76** | **16** | **24** | **24** |
| *Develop a “zero harm” culture* | Public | Advocacy | **23** | **0,78** | **20** | **25** | **17** |
| *Transport services to phone ahead of time to let patients know that they will be arriving to pick them up* | Other services | Organization of care | **24** | **0,76** | **15** | **7** | **30** |
| *If change of carer needs to occur, the new carer is introduced slowly* | Carers | Organization of care | **25** | **0,71** | **25** | **21** | **26** |
| *Provide a dementia friendly oven* | Home environment | Safety proofing | **26** | **0,73** | **28** | **23** | **20** |
| *Dismiss carers who do not have the correct values despite the training* | Carers | Recruitment and vetting | **27** | **0,73** | **27** | **26** | **18** |
| *Try to provide option for familiar family faces to stay in the hospital with the patient to decrease the patient’s deterioration and disorientation* | Carers | Organization of care | **28** | **0,63** | **26** | **28** | **28** |
| *Aim for two carers per patient to ensure consistency of care* | Carers | Organization of care | **29** | **0,63** | **29** | **29** | **27** |
| *Care agencies to use mobile tracking devices to monitor their staff* | Carers | Recruitment and vetting | **30** | **0,46** | **30** | **30** | **31** |
| *If continuity of care is not an option, strongly encourage the patient to move into a residential home* | Clinicians | Organization of care | **31** | **0,46** | **31** | **31** | **29** |

***AEA –average expert agreement; TPS – total priority score***
